# A new Egyptian Grid Weighted Mean Temperature (EGWMT) model using hourly ERA5 reanalysis data in GNSS PWV retrieval

**DOI:** 10.1038/s41598-024-64132-2

**Published:** 2024-06-25

**Authors:** Ragab Elhady Sleem, Mohamed Amin Abdelfatah, Ashraf El-Kutb Mousa, Gamal Saber El-Fiky

**Affiliations:** 1 Construction and Utilities Engineering Department, Faculty of Engineering, Zagazig University, Zagazig, Egypt; 2 Geodynamics Department, National Research Institute of Astronomy & Geophysics, Helwan, Egypt

**Keywords:** Weighted mean temperature, Precipitable water vapor, GNSS meteorology, ECMWF, Radiosonde, Atmospheric science, Environmental impact

## Abstract

Precise modeling of weighted mean temperature (*T*_*m*_) is essential for Global Navigation Satellite System (GNSS) meteorology. In retrieving precipitable water vapor (PWV) from GNSS, *T*_*m*_ is a crucial parameter for the conversion of zenith wet delay (ZWD) into PWV. In this study, an improved *T*_*m*_ model, named EGWMT, was developed to accurately estimate *T*_*m*_ at any site in Egypt. This new model was established using hourly ERA5 reanalysis data from European Centre for Medium-Range Weather Forecasts (ECMWF) covering the period from 2008 to 2019 with a spatial resolution of 0.25° × 0.25°. The performance of the proposed model was evaluated using two types of data sources, including hourly ERA5 reanalysis data from 2019 to 2022 and radiosonde profiles over a six-year period from 2017 to 2022. The accuracy of the EGWMT model was compared to that of four other models: Bevis, Elhaty, ANN and GGTm-Ts using two statistical quantities, including mean absolute bias (MAB) and root mean square error (RMSE). The results demonstrated that the EGWMT model outperformed the Bevis, Elhaty, ANN and GGTm-Ts models with RMSE improvements of 32.5%, 30.8%, 39% and 48.2%, respectively in the ERA5 data comparison. In comparison with radiosonde data, the EGWMT model achieved RMSE improvements of 22.5%, 34%, 38% and 19.5% against Bevis, Elhaty, ANN and GGTm-Ts models, respectively. In order to determine the significance of differences in means and variances, statistical tests, including t-test and F-test, were conducted. The results confirmed that there were significant differences between the EGWMT model and the four other models.

## Introduction

Water vapor, which considered an important component of the Earth’s atmosphere, has a major influence on the climate variations and water cycle in nature. Measuring water vapor accurately and tracking its distribution changes is a fundamental challenge in weather forecasting^1^. Precipitable water vapor (PWV) is the most common indicator for quantifying atmospheric water vapor. PWV is defined as the height of water column produced by the condensation of all tropospheric atmospheric water vapor in a given column per unit bottom area at any given time, into liquid water^2^. Conventional techniques for detecting water vapor involve water vapor radiometers, radiosondes, and remote sensing. The primary reasons for the limitations of these techniques are the high cost of equipment, extensive workloads, and low spatiotemporal resolution^3^.

In recent times, the Global Navigation Satellite System (GNSS) has become a significant method for obtaining PWV with high spatiotemporal resolution^4^. The GNSS signal traveling through the Earth's atmosphere is affected by refraction in the troposphere layer, causing a zenith delay known as Zenith Tropospheric Delay (ZTD). ZTD consists of two components: Zenith Hydrostatic Delay (ZHD) and Zenith Wet Delay (ZWD)^5^. One of the crucial steps in retrieving water vapor using GNSS technique is the conversion of ZWD into PWV^6^. This conversion is dependent on a conversion factor (II), which is determined by the weighted mean temperature of the atmosphere (*T*_*m*_). This technique has many advantages, including high precision, near real-time results, and high spatial and temporal resolution.

The weighted mean temperature (*T*_*m*_) can be calculated by the conventional method that requires the temperature and water vapor pressure vertical profiles^7^. The value of *T*_*m*_ at a specific location, such as a GNSS station, can be calculated by integrating temperature and humidity profiles obtained from either radiosonde data or atmospheric reanalysis products^8^. In many cases, it is not possible to obtain radiosonde data from a location that corresponds to the calculated point due to the limited number of radiosonde stations and the fixed launch schedule of the radiosonde balloon, which only occurs twice per day at UTC 0:00 and 12:00. Furthermore, since the reanalysis products are typically updated only once a month, they cannot be utilized for real-time *T*_*m*_ determination. Consequently, there is a requirement for a precise method of *T*_*m*_ calculation that can improve its efficiency and provide users with greater convenience^9^.

Bevis et al.^10^ first introduced the idea of GNSS meteorology. They developed a method for calculating the weighted mean temperature (*T*_*m*_), which is a crucial parameter for determining the amount of water vapor in the atmosphere using GNSS measurements. By subtracting ZHD from ZTD, ZWD can be computed, which allows for the accurate detection of atmospheric water vapor. They used a linear regression method between *T*_*m*_ and surface temperature (*T*_*s*_) to establish the model. This model was established using 8718 radiosonde profiles at 13 radiosonde stations in North America with a period of two years. This linear model can be expressed as, *T*_*m*_ = 70.2 + 0.72 *T*_*s*_, with a root mean square error (RMS) of 4.74K. Although the Bevis model is a regional model in U.S. continent, it was used as a global model. This model is considered the reference model and used to validate other regional and global models.

Many scholars have conducted a lot of research to establish global or regional weighted mean temperature models. For the global view, Ross and Rosenfeld^11^ conducted a study on *T*_*m*_ based on meteorological data from 53 radiosonde stations over a 23-year period. They established a linear relationship between *T*_*m*_ and $T_{s}$ to accurately estimate *T*_*m*_ and thus PWV from ground-based GNSS delays. Yao et al.^12^ built a new global weighted mean temperature (GWMT) model that directly uses three-dimensional coordinates and the day of year to calculate *T*_*m*_. This model was created using data from 135 radiosonde stations from 2005 to 2009 provided by Integrated Global Radiosonde Archive (IGRA). Yao et al.^13^ conducted a study to investigate the correlation between *T*_*m*_ and $T_{s}$ on a global scale based on the Global Geodetic Observing System (GGOS) atmosphere *T*_*m*_ data and ECMWF data. The data used for establishing this new model are the global 2° × 2.5° *T*_*m*_ and $T_{s}$ data from 2005 to 2011. Yao et al.^14^ presented a new global *T*_*m*_ model called GTM-III, which incorporates the variations in *T*_*m*_ on a semi-annual and diurnal basis using *T*_*m*_ grid data from 2005 to 2011 provided by GGOS atmosphere. Chen and Yao^15^ developed an empirical global *T*_*m*_ model named GTM_X. This model was established using ECMWF reanalysis data at 1° × 1° resolution from 2011 to 2013.

Huang et al.^3^ established a global grid empirical *T*_*m*_ model named GGTm using an 8-year period from 2007 to 2014 provided by GGOS. Li et al.^16^ proposed a global grid-based *T*_*m*_ model named GTm-R using ECMWF data from 2011 to 2017 with a horizontal resolution of 1° × 1° every 6 h. Sun et al.^17^ established a global grid-based *T*_*m*_ model named GGNTm with a horizontal resolution of 1° × 1° using ERA5 monthly mean reanalysis data from 2008 to 2017. Yang et al.^18^ proposed a global *T*_*m*_ model named GGTm-Ts using GGOS atmosphere *T*_*m*_ and ECMWF data from 2011 to 2015. This global 2.5° × 2° grid data model can provide *T*_*m*_ at any site in two modes. The first mode is in the case of measured $T_{s}$, i.e., the accurate mode; the other mode is for the case of $T_{s}$ provided by a subroutine, i.e., the normal mode. The new model was compared to three other models using the GGOS atmosphere and radiosonde stations in 2016. The results indicated that the GGTm-Ts model in the accurate mode performed well compared to the other models. Huang et al.^19^ presented a global high-precision *T*_*m*_ grid model named GGTm-H using data from Modern-Era Retrospective Analysis for Research and Applications, version-2 (MERRA-2), with a horizontal resolution of 0.5° × 0.625° every 6 h.

For the regional view, Liou et al.^20^ established a linear regional model in Taipei, Taiwan, which relates *T*_*m*_ with $T_{s}$ in linear regression using 10 years of radiosonde data. Bokoye et al.^21^ conducted a regional model for weighted mean temperature in Canada and Alaska. Suresh Raju et al.^22^ conducted a regional model in India for *T*_*m*_ based on $T_{s}$ from eight India meteorological department (IMD) stations in period of three years. Boutiouta and Lahcene^23^ made a study in Algeria and proposed a method to develop a regional model for *T*_*m*_ by the observations of Algerian radiosonde stations for a period of three years (from 2005 to 2007). Sapucci^24^ developed a regional *T*_*m*_ model in Brazil using statistical analysis of 90,000 radiosonde profiles at 12 Brazilian stations during a period from 1961 to 1993. Mekik and Deniz^25^ developed a regional model for *T*_*m*_ in Turkey using observations of eight Turkish radiosonde stations in 2011. Maghrabi et al.^26^ developed a simple empirical *T*_*m*_ model in Saudi Arabia using the observations of 107,776 profiles from seven radiosonde stations during the period from 1985 to 2016. Wang et al.^27^ established a regional linear regression $T_{m}$ model for Antarctic and Arctic using radiosonde profiles of 12 stations in Antarctic and 58 stations in Arctic from 2008 to 2015.

Wang et al.^28^ analyzed the region-specific and weather characteristics of the linear relationship between *T*_*m*_ and $T_{s}$ using 860,054 radiosonde profiles. These profiles were from 88 radiosonde stations in China from 2005 to 2018 and were used to establish a new *T*_*m*_ model for china which achieved the linear relationship between *T*_*m*_ and $T_{s}$. Zhang et al.^29^ proposed a new *T*_*m*_ model for Greenland using ERA5 reanalysis data from 1990 to 2018, and a data-driven principal component analysis (PCA) method. Saxena and Dwivedi^30^ developed a novel site specific *T*_*m*_ model ($T_{m - SSM}$) in India using ERA5 reanalysis data from 2016 to 2018 for 13 CORS (continuously operating reference stations) in the Survey of India (SOI) network. Yang et al.^31^ used ERA5 reanalysis data from 2001 to 2010 to obtain two sets of grid coefficients with a spatial resolution of 1° × 1° to accurately compute *T*_*m*_ over China using GPT3 (Global Pressure and Temperature 3) model. GPT3 model, presented by Landskron and Böhm^32^, is an empirical model that calculates meteorological parameters on a 5° × 5° as well as a 1° × 1° global grid.

In Egyptian region, Elhaty et al.^33^ utilized 3600 profiles from four radiosonde stations in Egypt from 2015 to 2016 to establish a new *T*_*m*_ model for Egypt. This regional model was evaluated against other six models, namely Bevis, Schueler, Yao, Liou, Suresh Raju and Wayan. The results indicated that the new model’s performance was slightly improved compared to the other models. Abdelfatah^34^ utilized a new approach to develop a regional *T*_*m*_ model in Egypt. The model, known as ANN, was created using machine learning technology and based on 2500 vertical profiles obtained from six radiosonde stations in 2017.

In this study, a new Egyptian Grid Weighted Mean Temperature (EGWMT) model is developed to accurately compute *T*_*m*_ at any location in Egypt. The new model was established using ERA5 reanalysis data from 2008 to 2019, with a spatial resolution of 0.25° × 0.25° and a high temporal resolution of 1 h. The model took into account the vertical lapse rate of *T*_*m*_ and calculated it at each grid point. To validate this new model, two types of data sources were utilized. The first type is ERA5 reanalysis data from 2019 to 2022, with grids of 0.25° × 0.25° and a 1-h time interval. The second type of data is radiosonde measurements collected from five radiosonde stations in Egypt over a six-year period from 2017 to 2022. In order to objectively assess the accuracy of the new model, it is compared with Bevis, Elhaty, ANN and GGTm-Ts models under the same conditions.

## Data and methods

### ERA5 reanalysis products

ERA5 reanalysis products are a collection of global atmospheric reanalysis data provided by the European Centre for Medium-Range Weather Forecasts (ECMWF). These products can give information about various meteorological parameters. The ERA5 reanalysis covers the period from 1979 to the present, and new data is continually added with a delay of 5 days. The data is presented in a gridded format with a high temporal resolution of 1 h and a spatial resolution of 0.25° × 0.25°. The pressure-level products divide the atmosphere into 37 vertical layers based on pressure, ranging from 1,000 to 1 hectopascal (hPa). These products also offer meteorological data for each layer's surface^35^.

In this study, ERA5 reanalysis hourly data on pressure levels are used with a spatial resolution of 0.25° × 0.25° and a high temporal resolution of 1 h. The study area covers the entire region of Egypt, from 22°N to 32°N and from 25°E to 37°E. The ERA5 data can be downloaded from the website: https://cds.climate.copernicus.eu/cdsapp#!/home^36^. The ERA5 pressure-level products from 2008 to 2022, including geopotential, temperature, relative humidity, and pressure, are utilized to obtain the *T*_*m*_ for modeling and validation purposes. The ERA5 products from 2008 to 2019 are employed to establish the model, while the products from 2020 to 2022 are utilized for validation.

### Radiosonde measurements

**Figure 1:**
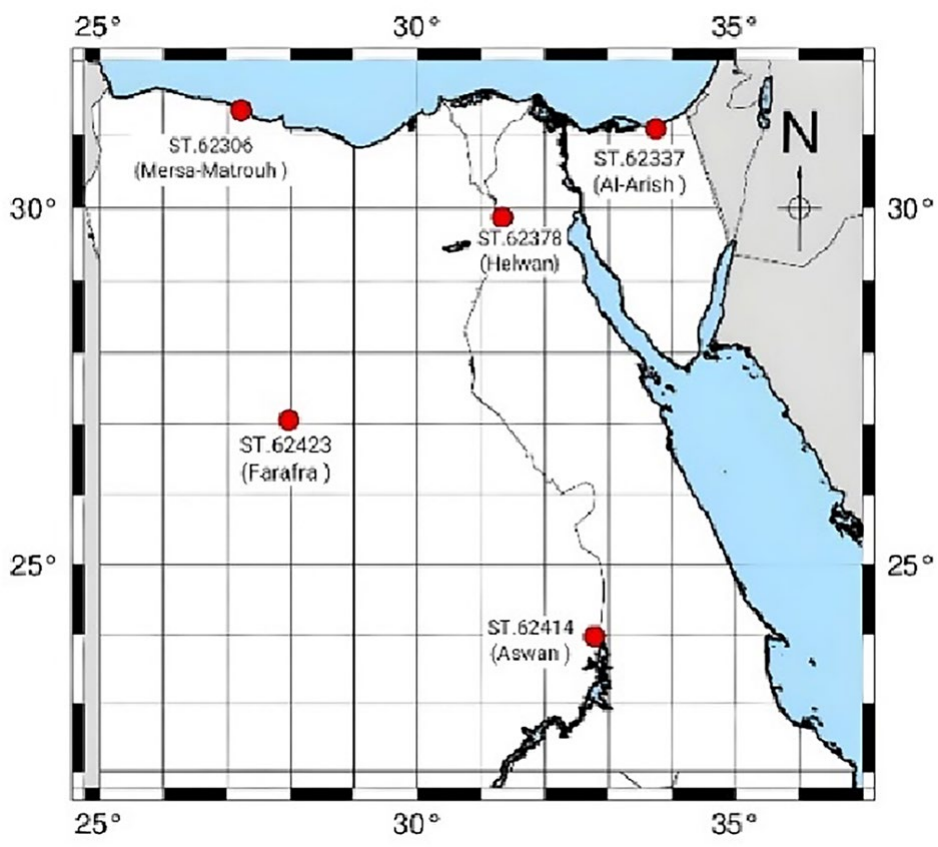
The distribution of radiosonde stations in Egypt.

Radiosonde measurements play a crucial role in assessing other weather observations and model predictions. These data are collected daily at 00:00 and 12:00 UTC, with a time interval of 12 h. Radiosonde measurements provide meteorological profile including pressure, temperature, relative humidity, and wind speed, at specific pressure levels. In this study, the meteorological data from five radiosonde stations in Egypt were collected over a six-year period from 2017 to 2022 to validate the EGWMT model. These radiosonde stations are located in Egypt, as shown in Fig. 1. The radiosonde data is from the Integrated Global Radiosonde Archive (IGRA), which is a newly released radiosonde data set from the National Oceanic and Atmospheric Administration (NOAA) National Climatic Data Centre. This data can be downloaded from the website: https://ruc.noaa.gov/raobs/^37^.

### T_m_ calculation

The weighted mean temperature (*T*_*m*_) is a crucial parameter to calculate the conversion factor (II), which is very important in retrieving perceptible water vapor (PWV) using GNSS technique. The perceptible water vapor and the conversion factor can be calculated and expressed as follows:

1 $$PWV = ZWD*II$$

2 $$II = \frac{{10^{6} }}{{\rho_{w} R_{v} \left( {K_{2}^{{\prime }} \frac{{K_{3} }}{{T_{m} }}} \right)}}$$

where $\rho_{w}$ is the liquid water density ($\rho_{w}$ = $10^{3} {\text{ kg}}/{\text{m}}^{3}$), $R_{v}$ is the specific gas constant of water vapor ($R_{v}$ = 461.5 J/ (k.kg)), and $\mathop {K_{2} }\limits$ (22.1 k/hPa) and $K_{3}$ ($3.739 \times 10^{5} {\text{ k}}^{2} /{\text{hpa}}$) are the atmospheric refraction constants (e.g. ^25^,^38^).

The value of *T*_*m*_ at a specific location can be calculated by numerical integration method. The numerical integration is the most accurate method and easy to be implemented. The formula to calculate *T*_*m*_ can be expressed as follows:

3 $${\text{T}}_{m} = { }\frac{{\mathop \smallint \nolimits_{{h_{s} }}^{{h_{t} }} \frac{e}{T} dh}}{{\mathop \smallint \nolimits_{{h_{s} }}^{{h_{t} }} \frac{e}{{T^{2} }} dh}}$$

4 $${\text{T}}_{m} = { }\frac{{\mathop \sum \nolimits_{i = 1}^{N} \left( {\frac{{e_{i} }}{{T_{i} }} + \frac{{e_{i + 1} }}{{T_{i + 1} }}} \right)\frac{{\Delta h_{i} }}{2}}}{{\mathop \sum \nolimits_{i = 1}^{N} \left( {\frac{{e_{i} }}{{T_{i}^{2} }} + \frac{{e_{i + 1} }}{{T_{i + 1}^{2} }}} \right)\frac{{\Delta h_{i} }}{2}}}$$

where $e$ and ${\text{T}}$ are the water vapor pressure (hPa) and the atmospheric temperature (k) along the zenith direction, respectively, $h_{s}$ (m) is the height of the station and $h_{t}$ (m) is the height of the tropopause^39^. $\Delta h_{i}$ is the thickness of the atmospheric layer, and $e_{i}$ and $T_{i}$ are the water vapor pressure and temperature at the bottom of the atmospheric layer, respectively. $e_{i + 1}$ and $T_{i + 1}$ are the water vapor pressure and temperature at the top of the atmospheric layer, respectively, and N is the number of layers (e.g.^40^,^41^).

5 $$e = { }\frac{{RH . e_{s} }}{100}$$

6 $$e_{s} = { }6.11 \times 10^{{\left( {\frac{{7.5 \times T_{d} }}{{237.3 + T_{d} }}} \right)}}$$

where $RH$ is the relative humidity, $e_{s}$ (hPa) is the saturated vapor pressure, and $T_{d}$ is the atmospheric temperature (℃).

The *T*_*m*_ lapse rate is a significant parameter for the vertical adjustment of *T*_*m*_. *T*_*m*_ at the reference level can be converted to *T*_*m*_ at a target level through vertical adjustment using the concept of *T*_*m*_ lapse rate. The linear relationship between *T*_*m*_ and height can be used to describe the *T*_*m*_ lapse rate (e.g.^9^,^42^). This relationship can be expressed as follows:

7 $$T_{m} = \beta \times \left( {h - h_{0} } \right) + T_{m0}$$

where $\beta$ is the *T*_*m*_ vertical lapse rate (K/Km), $h$ denotes the target height (Km), $h_{0}$ is the reference height (Km), and $T_{m0}$ represents the weighted mean temperature at the reference level. This formula is used in this study to calculate the vertical lapse rate at each grid point.

### T_m_ Calculated with Empirical Models

The value of *T*_*m*_ at any site can be calculated using *T*_*m*_ empirical models, which is very important parameter in real-time retrieving GNSS-PWV from GNSS-ZWD. In recent years, many scholars have conducted a lot of research on *T*_*m*_ modeling to establish *T*_*m*_ empirical models. Most of the previous *T*_*m*_ models can be described by the following equation^41^:

8 $$T_{m} = T_{1} \left( {T_{s} } \right) + T_{2} \left( {doy} \right) + T_{3} \left( {doy} \right) + T_{4} \left( {hod} \right)$$

where $T_{1} \left( {T_{s} } \right)$ represents the *T*_*m*_ that calculated from surface meteorological data. $T_{2} \left( {doy} \right)$, $T_{3} \left( {doy} \right)$, and $T_{4} \left( {hod} \right)$ represent the annual, semiannual, and diurnal *T*_*m*_ variation components, respectively.

9 $$T_{1} \left( {T_{s} } \right) = A_{1} \times T_{s} + \varepsilon_{1}$$

10 $$T_{2} \left( {doy} \right) = A_{2} \cos \left( {\frac{DOY*2\pi }{{365.25}}} \right) + A_{3} \sin \left( {\frac{DOY*2\pi }{{365.25}}} \right) + \varepsilon_{2}$$

11 $$T_{3} \left( {doy} \right) = A_{4} \cos \left( {\frac{DOY*4\pi }{{365.25}}} \right) + A_{5} \sin \left( {\frac{DOY*4\pi }{{365.25}}} \right) + \varepsilon_{3}$$

12 $$T_{4} \left( {hod} \right) = A_{6} \cos \left( {\frac{hod*2\pi }{{24}}} \right) + A_{7} \sin \left( {\frac{hod*2\pi }{{24}}} \right) + \varepsilon_{4}$$

where $A_{1}$, $A_{2}$, $A_{3}$, $A_{4}$, $A_{5}$, $A_{6}$, $A_{7}$, $\varepsilon_{1}$, $\varepsilon_{2}$, $\varepsilon_{3}$ and $\varepsilon_{4}$ are the model coefficients.

Some previous *T*_*m*_ models are listed in Table (1). The abbreviations in Table 1 represent different measurements. $\varphi$ is the latitude, $\lambda$ is the longitude, $H$ is the geoid height, $H_{ell}$ is the ellipsoidal height, $doy$ is the day of year, $hod$ is the hour of day, $mjd$ is the modified Julian date and $T_{s}$ is the surface temperature.

**Table 1:** Main differences between some previous $T_{m}$ models.

Model	Input	Expression	Data source	References
Bevis	$T_{s}$	$T_{{\text{m}}} = T_{1}$	Radiosonde	^10^
Elhaty	$T_{s}$	$T_{{\text{m}}} = T_{1}$	Radiosonde	^33^
ANN	$\varphi ,\lambda ,H,doy,T_{s}$	$T_{{\text{m}}} = T_{1} + T_{2}$	Radiosonde	^34^
GGT_m_-T_s_	$\varphi ,\lambda ,T_{s}$	$T_{{\text{m}}} = T_{1}$	GGOS/ECMWF	^18^
GPT2w	$\varphi ,\lambda ,mjd$	$T_{{\text{m}}} = T_{2} + T_{3}$	ECMWF	^43^
GTm_R	$\varphi ,\lambda ,H_{ell} ,doy,hod$	$T_{{\text{m}}} = T_{2} + T_{3} + T_{4}$	ECMWF	^16^

Empirical *T*_*m*_ models, which only rely on site coordinates and time, are commonly used to estimate global *T*_*m*_ values in real-time. However, these models have been found to be less accurate compared to linear regression models^44^. Linear regression is a better option for achieving highly accurate *T*_*m*_ estimates. However, most linear regression models are developed for specific regions and are only suitable for local use. Existing global research on *T*_*m*_ estimation is either limited to sparse stations or is based on different latitude ranges^18^. Empirical models are unable to accurately estimate global *T*_*m*_ values and establish a reliable *T*_*m*_ – *T*_*s*_ relationship. Additionally, linear regression models are not suitable for real-time applications when temperature sensors are absent at GNSS stations or when in situ surface temperature cannot be transmitted in real time to a data processing center. This limitation can negatively impact the continuous operation of a real-time GPS-PWV remote sensing system^18^.

## Model establishment

The new Egyptian Grid Weighted Mean Temperature (EGWMT) model is developed based on a 12-year period from 2008 to 2019, with a 1-h interval and in grids of 0.25° × 0.25° in the region of Egypt. The ERA5 reanalysis products from European Centre for Medium-Range Weather Forecasts (ECMWF), which include geopotential, temperature, relative humidity, and pressure, in 37 pressure levels were utilized to obtain the *T*_*m*_ values for establishing the new model. The *T*_*m*_ value at each level is calculated as the *T*_*m*_ from that level to the topmost level, as shown in Eq. (4).

The *T*_*m*_ can be interpolated or extrapolated to the surface to the Earth considering the *T*_*m*_ lapse rate. To compute the surface values of *T*_*m*_, a linear relationship between *T*_*m*_ and height is assumed. The vertical lapse rate of *T*_*m*_ is calculated using the data from only the four bottom levels from the ground, which are selected to cover most of the troposphere^44^.

The model coefficients are determined at each grid point throughout the study area. The proposed *T*_*m*_ model, EGWMT, can compute the *T*_*m*_ value at any site in Egypt using latitude ($\varphi$), longitude ($\lambda$), surface temperature ($T_{s}$), day of year ($doy$), and hour of day ($hod$). The equation for this model can be expressed as follows:

13 $$\begin{aligned} T_{m} =\; & \alpha_{1} + \alpha_{2} T_{s} + \alpha_{3} \cos \left( {\frac{doy*2\pi }{{365.25}}} \right) \\ & \quad + \alpha_{4} \sin \left( {\frac{doy*2\pi }{{365.25}}} \right) + \alpha_{5} \cos \left( {\frac{doy*4\pi }{{365.25}}} \right) \\ & \quad + \alpha_{6} \sin \left( {\frac{doy*4\pi }{{365.25}}} \right) + \alpha_{7} \cos \left( {\frac{hod*2\pi }{{24}}} \right) \\ & \quad + \alpha_{8} \sin \left( {\frac{hod*2\pi }{{24}}} \right) \\ \end{aligned}$$

where $\alpha_{1}$, $\alpha_{2}$, $\alpha_{3}$, $\alpha_{4}$, $\alpha_{5}$, $\alpha_{6}$, $\alpha_{7}$, and $\alpha_{8}$ are the model coefficients. These model coefficients are computed at 0.25° × 0.25° considering the spatial variations. At each grid point, the eight coefficients are determined using the least squares adjustment, with known values of $T_{s}$, $doy$, $hod$, and $T_{m}$.

**Figure 2:**
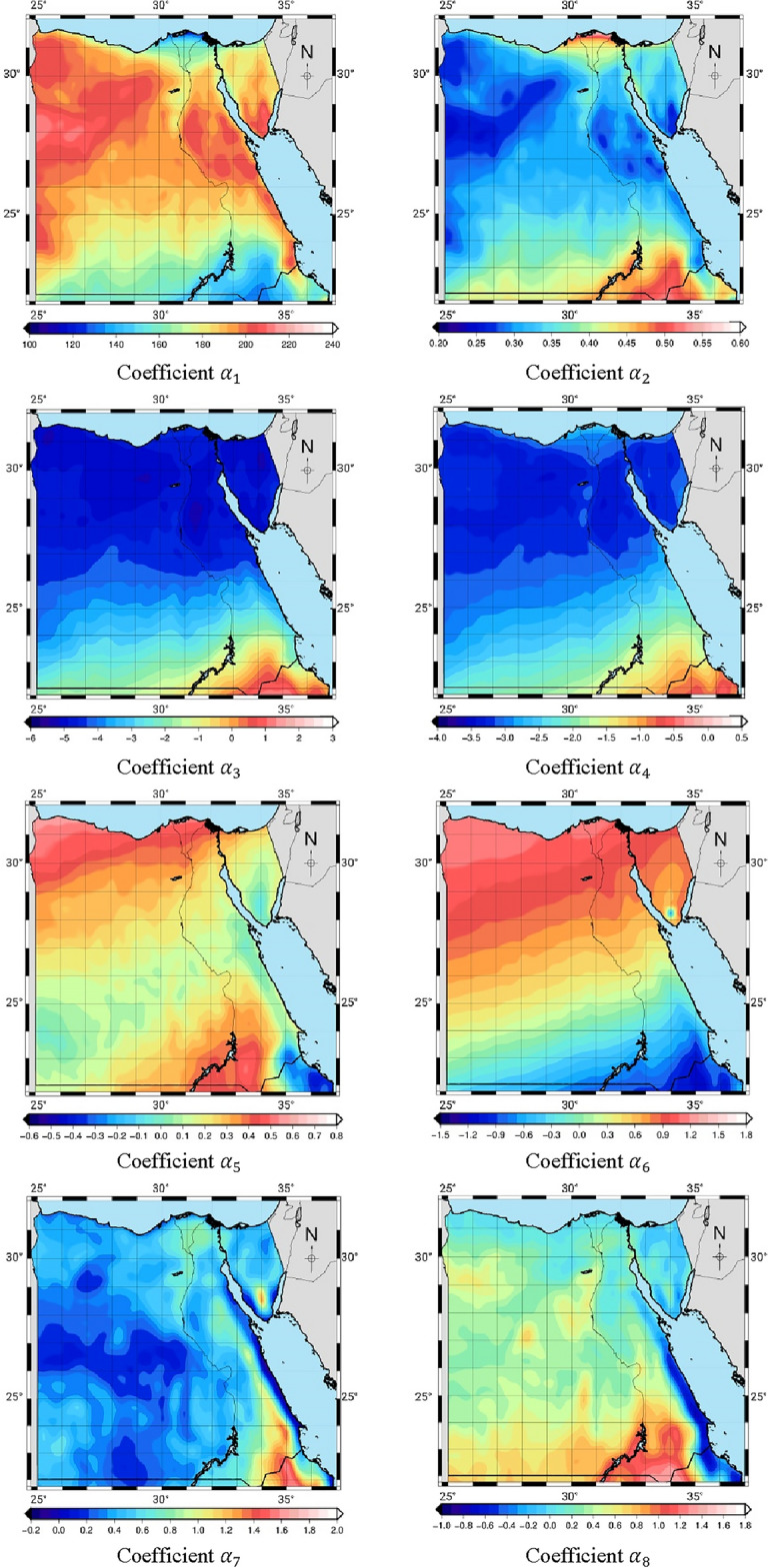
The eight coefficients for the EGWMT model.

Figure 2 shows the model coefficients $\alpha_{1}$, $\alpha_{2}$, $\alpha_{3}$, $\alpha_{4}$, $\alpha_{5}$, $\alpha_{6}$, $\alpha_{7}$, and $\alpha_{8}$ in grids of 0.25° × 0.25° in the region of Egypt. These eight coefficients are stored in the grid format. It is evident that these coefficients vary depending on the location. The latitude dependent is most evident in the eight coefficients. The $\alpha_{1}$ and $\alpha_{2}$ are the most essential coefficients. Generally, when $\alpha_{1}$ is large, $\alpha_{2}$ will be small. The value of weighted mean temperature at any site can be estimated using the coefficients, surface temperature and the observation time. Using the coordinates of the site, the values of the eight coefficients can be estimated and used as inputs in Eq. (13).

Based on the coordinates ($\varphi$ and $\lambda$) of a location and the observation time, the four nearest grid points will be selected, and their $T_{m}$ values will be calculated. Bilinear interpolation is then applied to estimate the $T_{m}$ value at the desired location.

## Statistical tests

### Statistical methods to evaluate the EGWMT model

To evaluate the performance of the newly developed $T_{m}$ model (EGWMT), $T_{m}$ values obtained from both ERA5 reanalysis data and radiosonde stations are selected as references. The two statistical quantities including mean absolute bias (MAB) and root mean square error (RMSE), are used to measure the accuracy of the EGWMT model results. The formulas for these quantities are shown in the following equations (e.g.^31^).

14 $${\text{MAB}} = { }\frac{1}{N}{ }\mathop \sum \limits_{i = 1}^{i = N} \left| {T_{mi}^{model} - T_{mi}^{Ref} } \right|$$

15 $${\text{RMSE}} = \sqrt {\frac{1}{N}{ }\mathop \sum \limits_{i = 1}^{i = N} (T_{mi}^{model} - T_{mi}^{Ref} )^{2} } { }$$

where N is the total number of observations, $T_{mi}^{model}$ is the $T_{m}$ values calculated by the model, and $T_{mi}^{Ref}$ is the $T_{m}$ reference values derived from ERA5 reanalysis data or radiosonde profiles.

### Test of hypothesis for two sample means (t-test)

The t-test is a statistical test used to compare the means of two samples. In this case, the test has been applied to the results of model validation, specifically comparing the EGWMT model with the four other models. Two types of data sources were used: ERA5 reanalysis data and radiosonde measurements. The two hypotheses, including the null hypothesis ($H_{0}$) and the alternative hypothesis ($H_{a}$), are specified as follows:

16 $$\begin{aligned} & H_{0} : \mu_{1} = \mu_{2} \\ & H_{a} : \mu_{1} \ne \mu_{2} \\ \end{aligned}$$

The statistic of the test is:

17 $$t = \frac{{{\upmu }_{1} - {\upmu }_{2} }}{{\sqrt {\frac{{S_{1}^{2} }}{{N_{1} }} + \frac{{S_{2}^{2} }}{{N_{2} }}} }}$$

The null hypothesis is rejected where:

18 $$\left| t \right| > t_{{{\raise0.7ex\hbox{$\alpha $} \!\mathord{\left/ {\vphantom {\alpha 2}}\right.\kern-0pt} \!\lower0.7ex\hbox{$2$}}}}$$

where $$\mu_{1}$$ and $$\mu_{2}$$ are the sample means, N1 and N2 are the sizes of the samples, $$S_{1}^{2}$$ and $$S_{2}^{2}$$ are the sample variances, and $$t_{{{\raise0.7ex\hbox{$\alpha $} \!\mathord{\left/ {\vphantom {\alpha 2}}\right.\kern-0pt} \!\lower0.7ex\hbox{$2$}}}}$$ is the tabulated t-value at confidence level 95% (e.g.^45^).

### Test of hypothesis for the ratio of two sample variances (F-test)

The F-test compares the variances of two samples. This test has been implemented on the results of model validation. The null hypothesis ($H_{0}$) and the alternative hypothesis ($H_{a}$), are stipulated as follows:

19 $$\begin{aligned} & H_{0} : \frac{{S_{1}^{2} }}{{S_{2}^{2} }}{ } = { }1 \\ & H_{a} : { }\frac{{S_{1}^{2} }}{{S_{2}^{2} }}{ } \ne 1 \\ \end{aligned}$$

The test statistic is:

20 $$F = \frac{{S_{1}^{2} }}{{S_{2}^{2} }},\quad \left( {S_{1}^{2} > S_{2}^{2} } \right)$$

The null hypothesis is rejected in the region:

21 $$F > F_{{{\raise0.7ex\hbox{$\alpha $} \!\mathord{\left/ {\vphantom {\alpha 2}}\right.\kern-0pt} \!\lower0.7ex\hbox{$2$}}}}$$

where $$S_{1}^{2}$$ is the larger variance, $$S_{2}^{2}$$ is the smaller variance, F is the test statistic value and $$F_{{{\raise0.7ex\hbox{$\alpha $} \!\mathord{\left/ {\vphantom {\alpha 2}}\right.\kern-0pt} \!\lower0.7ex\hbox{$2$}}}}$$ is the tabulated F-value at confidence level 95% (e.g.^45^,^46^).

## Performance analysis of the EGWMT model

**Figure 3:**
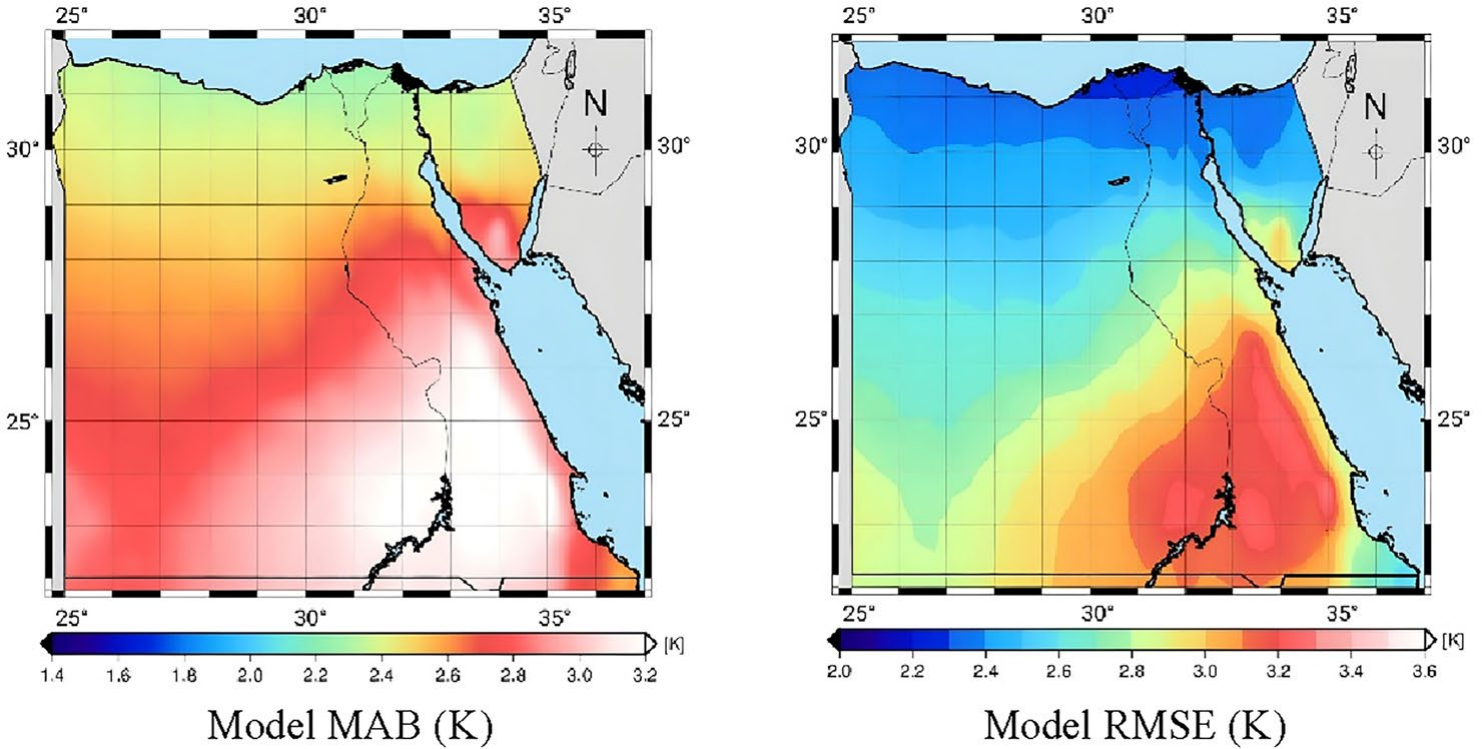
The EGWMT model performance.

The ERA5 products from 2008 to 2019 are employed to assess the performance of the EGWMT model, using two statistical methods to measure its accuracy at each grid point. These methods are MAB and RMSE, as shown in Fig. 3. The average value of model MAB is 2.12 K, while the minimum and maximum values of MAB are 1.71 K and 2.60 K, respectively. Whereas, the minimum and maximum values of RMSE are 2.20 K and 3.29 K, respectively, with an average value of 2.71 K.

**Figure 4:**
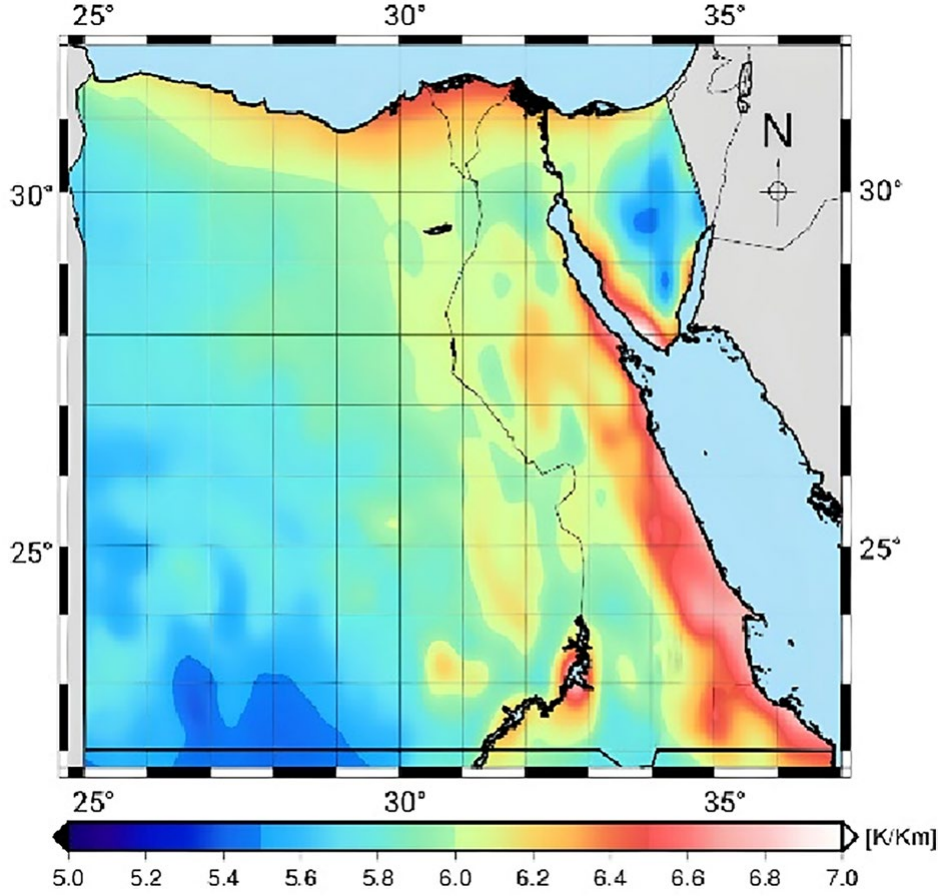
The average values of vertical $T_{m}$ lapse rates.

The average values of vertical $T_{m}$ lapse rates are calculated at all grid points in the region of Egypt, as shown in Fig. 4. It is shown that the average value of $T_{m}$ lapse rate is 6.05 K/Km. the minimum and maximum values of vertical $T_{m}$ lapse rates are 5.35 K/Km and 7.66 K/Km, respectively.

## Model validation

### Model validation using ERA5 reanalysis data

In order to objectively assess the performance of the EGWMT model, the $T_{m}$ reference values used for comparison were derived from ERA5 reanalysis grid data over a three-year period from 2020 to 2022. This new model was compared against four other models: Bevis, Elhaty, ANN, and GGTm-Ts. The Elhaty and ANN models are the only available local models for Egypt. The Bevis formula is considered the first proposed $T_{m}$ model and is regarded as the reference model for validating other regional and global models. The GGTm-Ts model is the latest available global model that covers the region of Egypt. Two statistical quantities are used to measure the accuracy of the EGWMT model at each grid point, MAB and RMSE.

**Figure 5:**
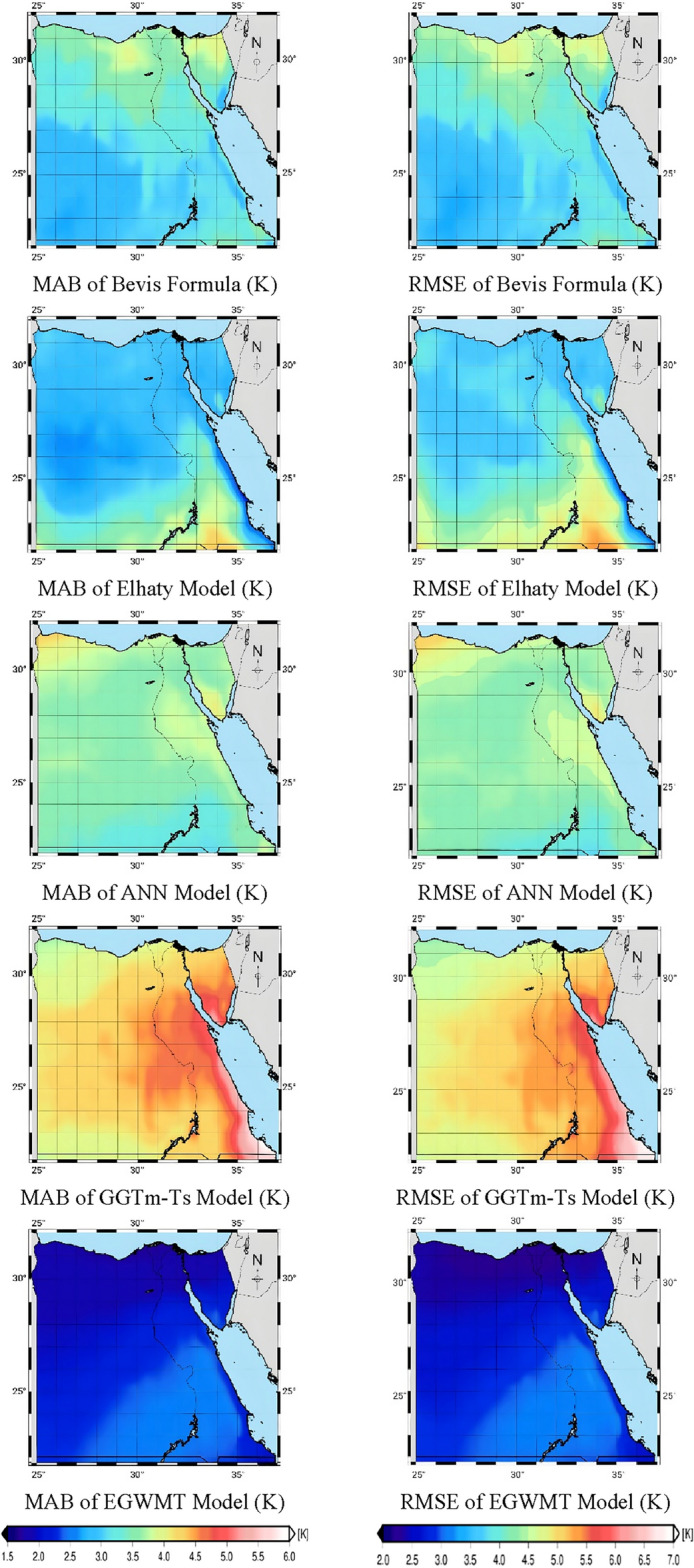
The distribution of MAB and RMSE for different models tested by ERA5 reanalysis data over a three-year period from 2020 to 2022.

The MAB and RMSE values of $T_{m}$ were calculated at each grid point for the five $T_{m}$ models. These values were stored in grids, as shown in Fig. 5. It is clear that the new model (EGWMT) performs well and achieves the smallest MAB and RMSE values. When comparing the four other models, the GGTm-Ts model performs the worst. This may be because the model is a global model and not specifically tailored to the region of Egypt. while the ANN model performs better than the GGTm-Ts model. Both Elhaty model and the Bevis formula achieve better results than the other two models. The Elhaty model slightly outperforms the Bevis formula.

Table 2 indicates the statistical results, including the minimum, maximum and mean values of the five models derived from ERA5 reanalysis data over a three-year period from 2020 to 2022. It shows that for EGWMT model, the average value of MAB is 2.14 K, the minimum and maximum values of MAB are 1.68 and 2.58, respectively. While, the RMSE is 2.70 K in average and ranges from 2.12 to 3.24 K. This new model performs well and achieves the lowest values of MAB and RMSE, because the EGWMT model is a regional model specialized for Egypt and may be due to the abundance of data used to establish the model in the Egypt region. It is developed based on hourly ERA5 reanalysis data for 12 years, taking into account temporal variations (day of year and hour of day) and spatial variations (in grids of 0.25° × 0.25°). The GGTm-Ts model performs worst and achieves the largest values of MAB and RMSE. It achieves 4.45 K in average and ranges from 3.57 to 6.10 in term of MAB. In term of RMSE, the average value is 5.20 K and ranging from 4.22 to 7.04 K. The ANN model performs better than the GGTm-Ts model and Elhaty model is better than the Bevis formula and the ANN model.

**Table 2:** Statistics of MAB and RMSE for different $T_{m}$ models derived from ERA5 reanalysis data.

Model	MAB (k)			RMSE (k)		
	Min	Max	Average	Min	Max	Average
Bevis	2.68	4.04	3.27	3.38	4.92	4.00
Elhaty	2.20	4.29	3.00	2.73	5.34	3.9
ANN	3.11	4.55	3.66	3.81	5.38	4.42
GGTm-Ts	3.57	6.10	4.45	4.22	7.04	5.20
EGWMT	1.68	2.58	2.14	2.12	3.24	2.70

To determine the significance of the differences between the means of the EGWMT model and the four other models derived from ERA5 reanalysis data, a t-test is conducted. The statistic of the t-test is shown in Eq. 17. The tabulated t-value is 1.96 at a confidence level 95%. The null hypothesis states that there is no significant difference between the $T_{m}$ means for the models. This hypothesis occurs when the values of the t-test are smaller than the tabulated t-value. The alternative hypothesis states that there is a significant difference between the $T_{m}$ means for the models. This hypothesis occurs when the values of the t-test are greater than the tabulated value. When comparing the EGWMT model to each of the four other models, the computed t-value is estimated. The computed values of the t-test when comparing the EGWMT model with Bevis formula, Elhaty, ANN, and GGTm-Ts models are 10.51, 8.20, 13.17 and 17.67, respectively. All of these values are greater than the tabulated t-value, which means that the null hypotheses can be rejected. This indicates that there are significant differences between the EGWMT model and the other models. Therefore, it can be concluded that the EGWMT model has the best performance.

The F-test is implemented to determine the significance of the variances between the EGWMT model and the four other models obtained from ERA5 reanalysis data. The F-test statistics are shown in Eq. 20. The tabulated value of F is 1.43 at a confidence level 95%. The null hypothesis is achieved when the F-test values are smaller than the tabulated value, while the alternative hypothesis is achieved when the values of F-test are larger than the tabulated value. The estimated F-values when comparing the EGWMT model with Bevis formula, Elhaty, ANN, and GGTm-Ts models are 2.20, 2.09, 2.69 and 3.73, respectively. The null hypotheses for all cases are rejected because all F values are greater than the tabulated F-value. This means that there are significant differences between the EGWMT model and the other models.

The seasonal variations in $T_{m}$ were derived from ERA5 reanalysis grid data from 2020 to 2022 in order to assess the seasonal performance of the EGWMT model. The MAB and RMSE values for the five models were calculated at each grid point for the four seasons to assess the EGWMT model and compare it to the four other models.

**Figure 6:**
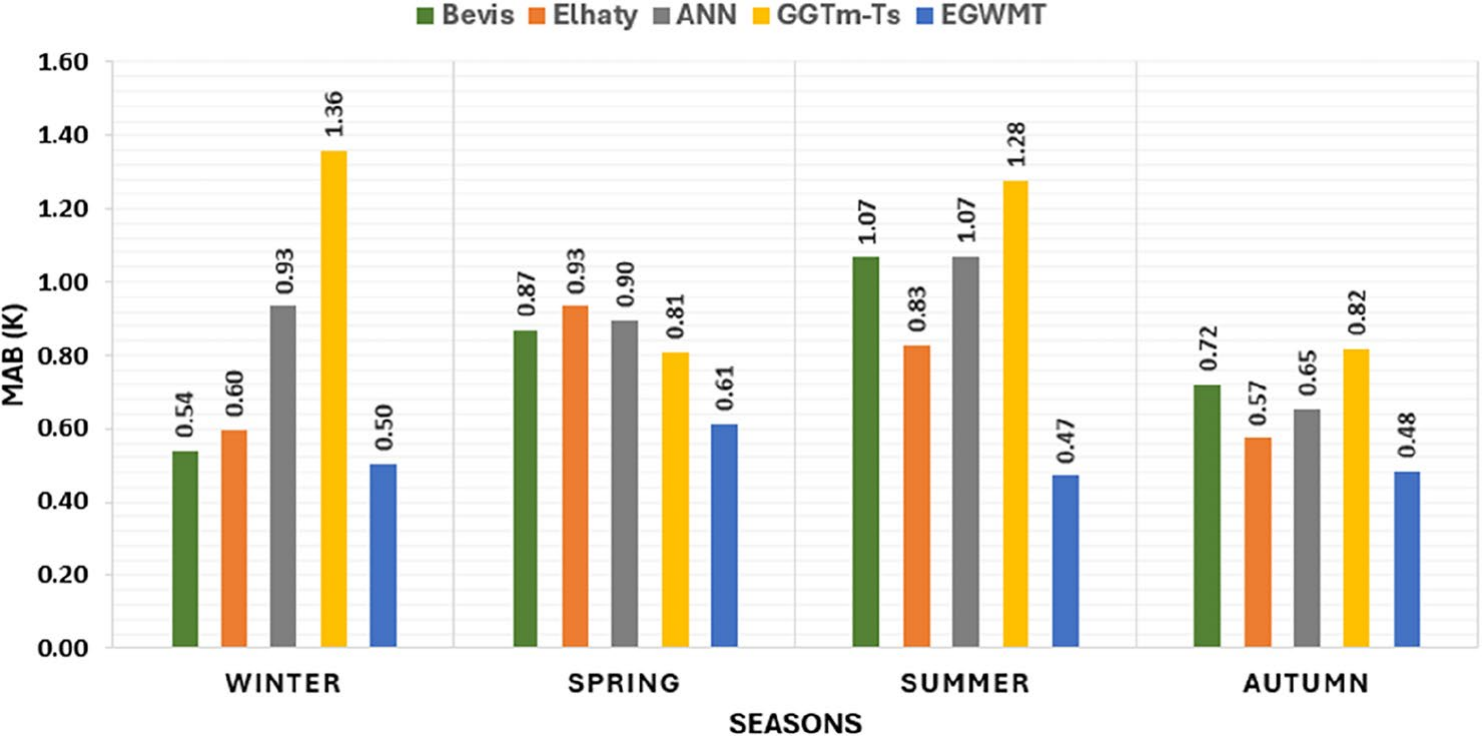
The average MAB of $T_{m}$ derived from the five models for the four seasons.

The bar chart in Fig. 6 demonstrates the average MAB of $T_{m}$ for the five models for each season. The results clearly indicate that the EGWMT model achieved the lowest values of $T_{m}$ MAB for all seasons. On the other hand, the GGTm-Ts model performs the worst, with the largest values of $T_{m}$ MAB. The t-test here is implemented to determine the significance of differences between the means of the EGWMT model and the four other models. The tabulated t-value is 1.96 at a confidence level 95% as shown in Eq. 17. All values of computed t-test, when comparing the EGWMT model with the other models are greater than the tabulated t-value, except in the case of Bevis formula in Winter. In the case of comparing the EGWMT model with Bevis formula in winter, the computed t-test is 0.92, which is smaller than the tabulated t-value. Therefore, the null hypotheses can be rejected for all cases except Bevis formula in Winter. This means that there are significant differences between the EGWMT model and the other models except for the Bevis formula in Winter.

**Figure 7:**
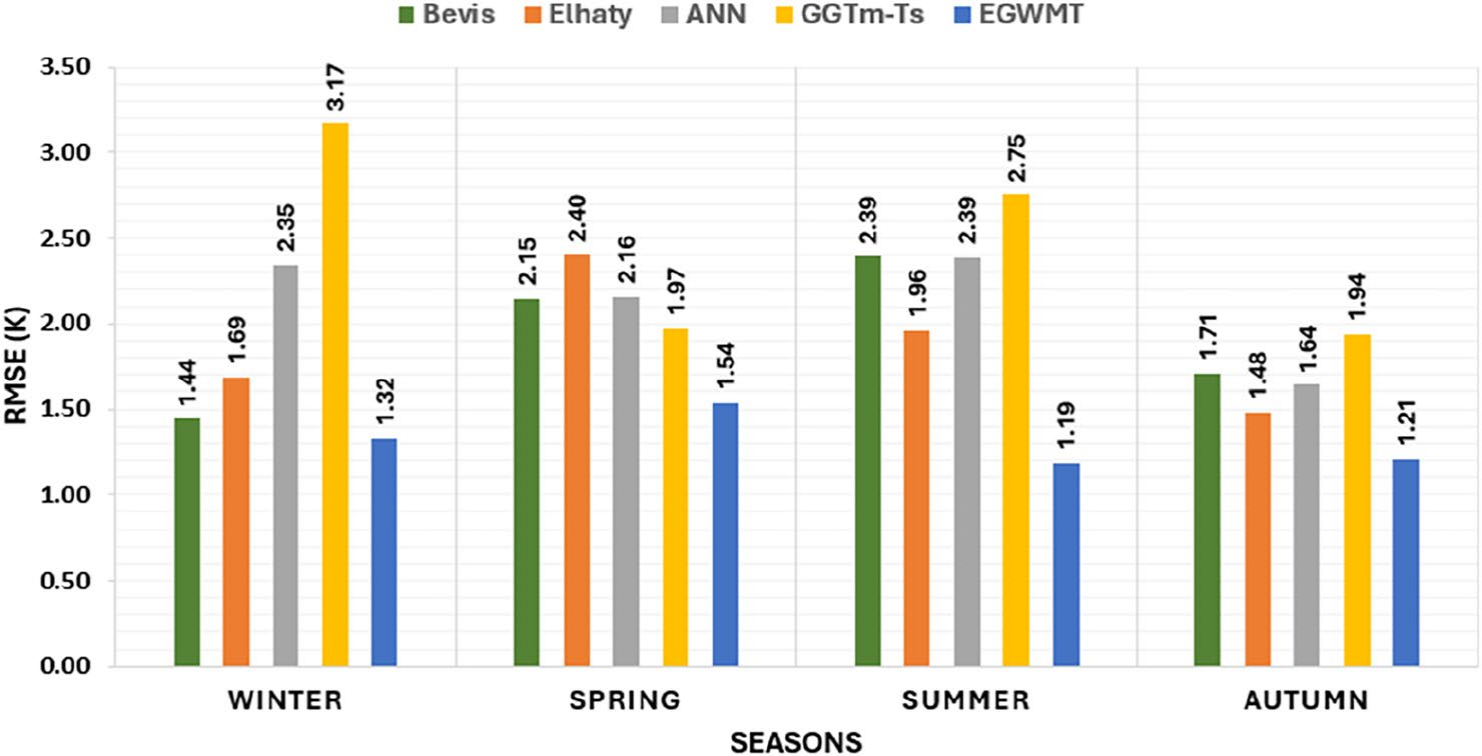
The average RMSE derived from the five models for the four seasons.

The bar chart in Fig. 7 illustrates the average RMSE for the five models for each season. The EGWMT model consistently demonstrates the best performance, achieving the smallest values of RMSE for all seasons. Here, the F-test is conducted to determine the significance of the variances between the EGWMT model and the four other models as shown in Eq. 20. The tabulated F-value is 1.43 at a confidence level 95%. All estimated F-test values, when comparing the EGWMT model with the other models are greater than the tabulated F-value, except in the case of Bevis formula in Winter, where the computed F-value is 1.18. This means that there are significant differences between the EGWMT model and the other models, except for the Bevis formula in Winter.

### Model validation using radiosonde data

The radiosonde measurements at any given site provide meteorological observations, including temperature, pressure, relative humidity, and wind speed at the surface, as well as at various pressure levels. The radiosonde observations from five radiosonde stations in Egypt were used over a six-year period from 2017 to 2022 to assess the EGWMT model and compare it to four other models. The $T_{m}$ values of each radiosonde station over a six-year period were calculated using the integration method and were considered as the reference values to validate the EGWMT model. The performance of the new model is assessed using two statistical quantities: MAB and RMSE.

**Figure 8:**
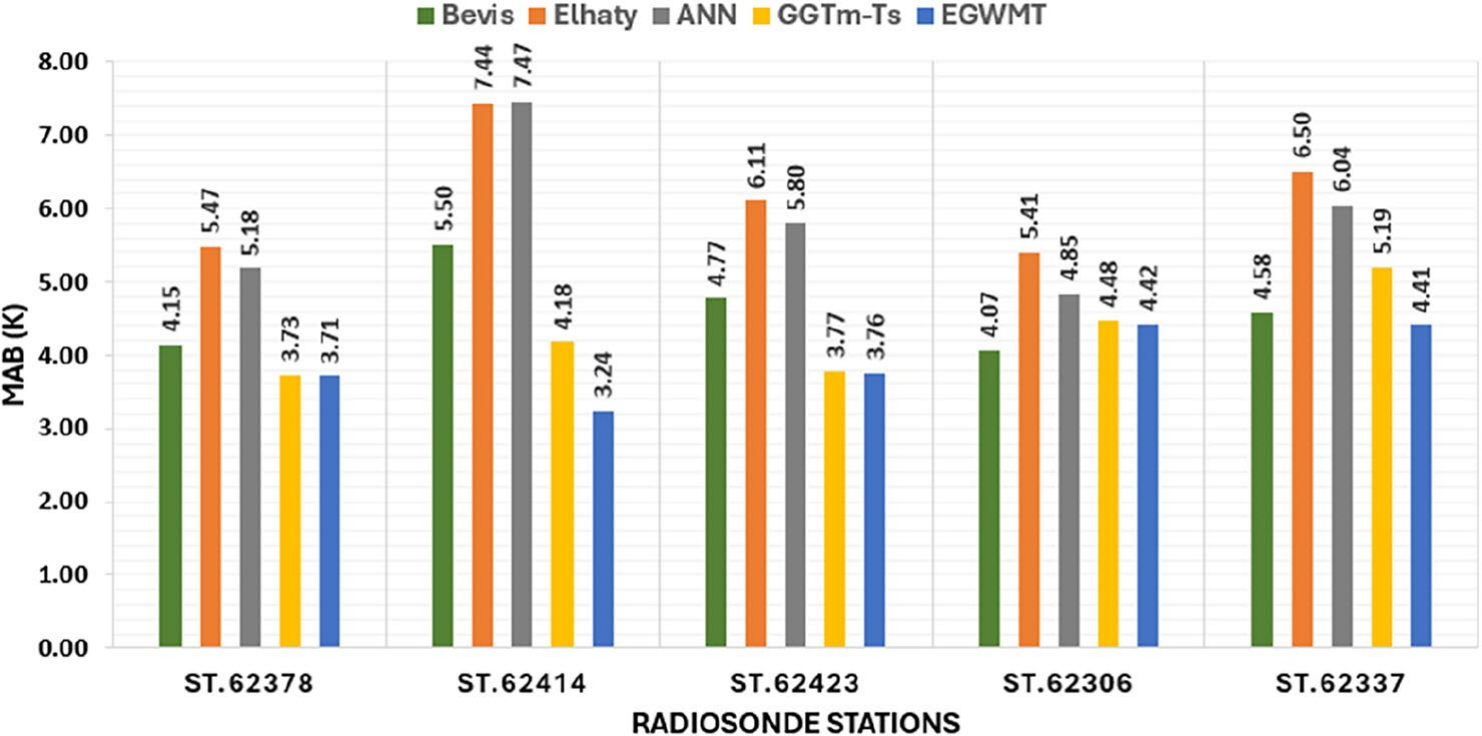
The average MAB of $T_{m}$ derived from the five models at five radiosonde stations in Egypt.

The bar chart in Fig. 8 illustrates the average $T_{m}$ MAB for the five models at each radiosonde station. It is clear that the new model (EGWMT) achieved the lowest values of MAB at most radiosonde stations, except at station 62,306 (Mersa-Matrouh). At this station, the Bevis formula achieved the lowest value of MAB of 4.07 K. The MAB of the GGTm-Ts model are closest to those of the EGWMT model. The performance of GGTm-Ts model is better than that of the Bevis formula in most stations. The mean values of MAB of all radiosonde stations are 4.55 K for Bevis, 6.03 K for Elhaty, 5.71 K for ANN, 4.14 K for GGTm-Ts, and 3.89 K for EGWMT model. The EGWMT model achieves the lowest MAB value. Additionally, the performance of the Bevis formula is better than that of the ANN and Elhaty models.

The t-test here is implemented to determine the difference significance between the means of the EGWMT model and the four other models derived from radiosonde measurements. The t-test statistics are shown in Eq. 17. The tabulated value of t at a confidence level 95% is 1.96. When the values of the t-test are smaller than the tabulated t-value, the null hypothesis is accepted. However, when these values are greater than the tabulated value, the null hypothesis is rejected. When the EGWMT model is compared with Bevis formula, Elhaty, ANN, and GGTm-Ts models, the estimated t-test values are 10.37, 30.06, 23.60 and 4.08, respectively. The t-test values for all of these cases are larger than the tabulated value, indicating that the null hypotheses are rejected. This implies that there are significant differences between the EGWMT model and the four other models.

**Figure 9:**
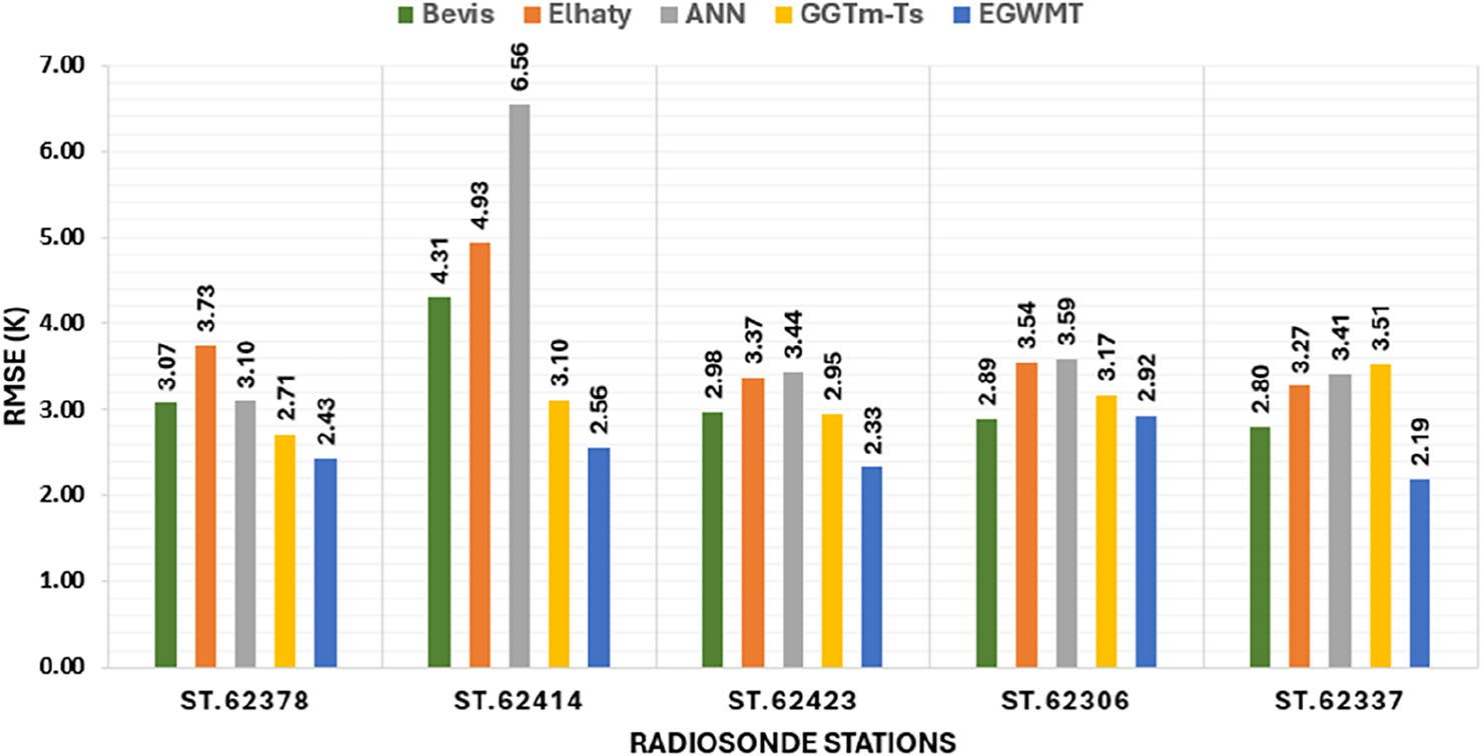
The average RMSE for the five models at five radiosonde stations in Egypt.

The bar chart in Fig. 9 shows the average RMSE for the five models at each radiosonde station. At most stations, the EGWMT model has the smallest RMSE, and The ANN model has the largest RMSE. The GGTm-Ts model has smaller RMSE than the Bevis formula at most stations. The mean values of RMSE of all radiosonde stations are 3.33 K for Bevis, 3.95 K for Elhaty, 4.39 K for ANN, 3.05 K for GGTm-Ts, and 2.65 K for EGWMT models. Generally, the EGWMT model shows the highest accuracy with the smallest value of mean RMSE, and its ranges are smaller than that of the other models.

In order to determine the significance of the variances between the EGWMT model and the four other models derived from radiosonde measurements, the F-test is conducted. The tabulated F-value is 1.43 at a confidence level 95%. The null hypothesis is accepted when the F-test values are smaller than the tabulated F-value. The estimated values of F-test when comparing the EGWMT model with Bevis formula, Elhaty, ANN, and GGTm-Ts models are 1.58, 2.22, 2.74 and 1.33, respectively. The F-values for all of these cases are greater than the tabulated F-value, indicating that the null hypotheses are rejected, except in the case of the EGWMT model with GGTm-Ts model. In this case, the estimated F-value is smaller than the tabulated value, which means that the null hypothesis can be accepted, and there is no significant difference between the variances of the EGWMT and GGTm-Ts models.

## Impact of ${\varvec{T}}_{{\varvec{m}}}$ on PWV retrieval

The goal of estimating $T_{m}$ is to convert the ZWD into GNSS-PWV by applying a conversion factor ($II$). Most of the GNSS stations are not equipped with meteorological sensors thus, it is difficult to conduct a comprehensive assessment of the impact of $T_{m}$ on GNSS-PWV. The errors in $T_{m}$ will indirectly affect the accuracy of GNSS-PWV estimation. Therefore, many studies have been conducted to investigate the impact of $T_{m}$ on the resultant GNSS-PWV (e.g.^47^,^44^). In this study, the impact of $T_{m}$ values from the EGWMT model and the four other models on PWV are analyzed using the same data as utilized in section "Model validation using ERA5 reanalysis data". The commonly used formula of the RMS between $T_{m}$ and PWV can be expressed as:

22 $$\frac{{RMS_{PWV} }}{PWV} = \frac{{RMS_{II} }}{II} = \frac{{K_{3} \cdot RMS_{{T_{m} }} }}{{\left[ {\frac{{K_{3} }}{{T_{m} }} + K_{2}^{{\prime }} } \right]T_{m}^{2} }} = \frac{{K_{3} }}{{\left[ {\frac{{K_{3} }}{{T_{m} }} + K_{2}^{{\prime }} } \right]T_{m} }} \cdot \frac{{RMS_{{T_{m} }} }}{{T_{m} }}$$

where $RMS_{PWV}$ represents the error of PWV, $RMS_{II}$ is the error of conversion factor ($II$), and $RMS_{{T_{m} }}$ denotes the error of $T_{m}$. $\mathop {K_{2} }\limits$ (22.1 k/hPa) and $K_{3}$ (3.739 × 10^5^ k^2^/hpa) are the atmospheric refraction constants. $RMS_{PWV} /PWV$ denotes the relative error of PWV^48^.

Table 3 presents the statistical results (Min, Max, and Average) of the errors in PWV $\left( {RMS_{PWV} } \right)$ and the relative errors in PWV $\left( {RMS_{PWV} /PWV} \right)$ for the five models. The results clearly indicate that the EGWMT model outperforms the four other models in terms of both $RMS_{PWV}$ and $RMS_{PWV} /PWV$. The average values of $RMS_{PWV}$ and $RMS_{PWV} /PWV$ obtained from the EGWMT model are 0.16 mm and 0.88%, respectively, which are the lowest values compared to other models. This may be attributed to that the EGWMT model is a regional model specifically established for Egypt and benefits from the abundance of data used to establish it.

**Table 3:** Statistics of errors and relative errors in PWV for different models.

Model	$RMS_{PWV} /PWV$(%)			$RMS_{PWV}$(*mm*)		
	Min	Max	Average	Min	Max	Average
Bevis	1.37	1.55	1.45	0.05	0.76	0.25
Elhaty	1.30	1.47	1.37	0.05	0.73	0.24
ANN	1.48	1.67	1.56	0.06	0.82	0.27
GGTm-Ts	1.59	1.80	1.68	0.06	0.89	0.29
EGWMT	0.84	0.95	0.88	0.03	0.47	0.16

## Conclusions

Precipitable water vapor (PWV) is a crucial component of water cycle and weather forecasting. Conventional techniques for measuring PWV don’t meet the increasing demands of meteorological development. As a result, Global Navigation Satellite System (GNSS) has emerged as a significant method for obtaining PWV with high spatiotemporal resolution. In GNSS meteorology, ZWD can be converted to PWV using a conversion factor (II), which is determined by the weighted mean temperature ($T_{m}$). Therefore, it is very important to obtain a precise method for calculating $T_{m}$.

In this research, a new Egyptian Grid Weighted Mean Temperature (EGWMT) model was developed to accurately estimate $T_{m}$ at any location in Egypt. This model was established using ERA5 reanalysis data from European Centre for Medium-Range Weather Forecasts (ECMWF) from 2008 to 2019, with a spatial resolution of 0.25° × 0.25° and a high temporal resolution of 1 h. In order to validate and compare this new model with the Bevis, Elhaty, ANN and GGTm-Ts models, two types of data sources were utilized. The first type consisted of ERA5 reanalysis data from 2019 to 2022, with grids of 0.25° × 0.25° and a 1-h time interval. At each grid point, the values of MAB and RMSE were calculated for the five $T_{m}$ models. The estimated average values of MAB for the EGWMT, Bevis formula, Elhaty, ANN, and GGTm-Ts models were found to be 2.14 K, 3.27 K, 3.00 K, 3.66 K, and 4.45 K, respectively. The average values of RMSE were 4.00 K for Bevis, 3.90 K for Elhaty, 4.42 K for ANN, 5.20 K for GGTm-Ts, and 2.70 K for EGWMT model. Statistical tests, including t-test and F-test, were conducted to determine the significance of differences in means and variances. The results of these tests indicated that there were significant differences between the EGWMT model and the other models. Consequently, the new model (EGWMT) had the best results, achieving the smallest MAB and RMSE values.

The second type of data consisted of radiosonde profiles collected from five radiosonde stations in Egypt over a six-year period from 2017 to 2022. The average values of MAB were found to be 4.55 K for Bevis, 6.03 K for Elhaty, 5.71 K for ANN, 4.14 K for GGTm-Ts, and 3.89 K for EGWMT model. The average values of RMSE for the EGWMT, Bevis formula, Elhaty, ANN, and GGTm-Ts models were 2.65 K, 3.33 K, 3.95 K, 4.39 K, and 3.05 K, respectively. The EGWMT model achieved the smallest values of MAB and RMSE when compared to the other models. The t-test and F-test here were conducted to examine the significance of differences in means and variances. The results show that there were significant differences between the EGWMT model and the other models. Therefore, based on the obtained results, it is recommended to use the EGWMT model for estimating the $T_{m}$ at any site in Egypt as it performed the best among the other models.

## Data Availability

ERA5 reanalysis products provided by ECMWF are freely available and can be downloaded from: https://cds.climate.copernicus.eu/cdsapp#!/home. The radiosonde data from IGRA can be freely downloaded from: https://ruc.noaa.gov/raobs/. The datasets generated and analyzed during the current study are available from the corresponding author upon request.

## References

[1] Ning T (2016). Homogenized time series of the atmospheric water vapor content obtained from the GNSS reprocessed data. J. Clim..

[2] Wu M (2021). High-precision GNSS PWV and its variation characteristics in China based on individual station meteorological data. Remote Sens..

[3] Huang L, Jiang W, Liu L, Chen H, Ye S (2019). A new global grid model for the determination of atmospheric weighted mean temperature in GPS precipitable water vapor. J. Geod..

[4] Wang M (2023). The assessment and meteorological applications of high spatiotemporal resolution GPS ZTD/PW derived by precise point positioning. Acta Geodaetica et Cartographica Sinica.

[5] Chen P, Yao W, Zhu X (2014). Realization of global empirical model for mapping zenith wet delays onto precipitable water using NCEP re-analysis data. Geophys. J. Int..

[6] Askne J, Nordius H (1987). Estimation of tropospheric delay for microwaves from surface weather data. Radio Sci..

[7] Sun Z, Zhang B, Yao Y (1893). A global model for estimating tropospheric delay and weighted mean temperature developed with atmospheric reanalysis data from 1979 to 2017. Remote Sens..

[8] Hagemann S, Bengtsson L, Gendt G (2003). On the determination of atmospheric water vapor from GPS measurements. J. Geophys. Res. Atmos..

[9] Zhang H (2017). GPS PPP-derived precipitable water vapor retrieval based on Tm/Ps from multiple sources of meteorological data sets in China. J. Geophys. Res. Atmos..

[10] Bevis M (1992). GPS meteorology: Remote sensing of atmospheric water vapor using the global positioning system. J. Geophys. Res. Atmos..

[11] Ross RJ, Rosenfeld S (1997). Estimating mean weighted temperature of the atmosphere for Global Positioning System applications. J. Geophys. Res. Atmos..

[12] Yao Y, Zhu S, Yue S (2012). A globally applicable, season-specific model for estimating the weighted mean temperature of the atmosphere. J. Geodesy.

[13] Yao Y, Zhang B, Xu C, Chen J (2014). Analysis of the global T m–T s correlation and establishment of the latitude-related linear model. Chin. Sci. Bull..

[14] Yao Y, Xu C, Zhang B, Cao N (2014). GTm-III: A new global empirical model for mapping zenith wet delays onto precipitable water vapour. Geophys. J. Int..

[15] Chen, P., and Yao, W. GTm_X: A new version global weighted mean temperature model. In: *China Satellite Navigation Conference (CSNC) 2015 Proceedings*: Volume **II** (pp. 605–611). Springer. 10.1007/978-3-662-46635-3_51 (2015).

[16] Li Q, Yuan L, Chen P, Jiang Z (2020). Global grid-based T m model with vertical adjustment for GNSS precipitable water retrieval. GPS Solut..

[17] Sun P, Wu S, Zhang K, Wan M, Wang R (2021). A new global grid-based weighted mean temperature model considering vertical nonlinear variation. Atmos. Measurement Tech..

[18] Yang F (2023). GGTm-Ts: A global grid model of weighted mean temperature (Tm) based on surface temperature (Ts) with two modes. Adv. Space Res..

[19] Huang L (2023). A novel global grid model for atmospheric weighted mean temperature in real-time GNSS precipitable water vapor sounding. IEEE J. Sel. Top. Appl. Earth Obser. Remote Sens..

[20] Liou Y-A, Teng Y-T, Van Hove T, Liljegren JC (2001). Comparison of precipitable water observations in the near tropics by GPS, microwave radiometer, and radiosondes. J. Appl. Meteorol. Climatol..

[21] Bokoye A (2003). Multisensor analysis of integrated atmospheric water vapor over Canada and Alaska. J. Geophys. Res. Atmos..

[22] Suresh Raju C, Saha K, Thampi BV, Parameswaran K (2007). Empirical model for mean temperature for Indian zone and estimation of precipitable water vapor from ground based GPS measurements. Annales Geophysicae..

[23] Boutiouta S, Lahcene A (2013). Preliminary study of GNSS meteorology techniques in Algeria. Int. J. Remote Sens..

[24] Sapucci LF (2014). Evaluation of modeling water-vapor-weighted mean tropospheric temperature for GNSS-integrated water vapor estimates in Brazil. J. Appl. Meteorol. Climatol..

[25] Mekik C, Deniz I (2017). Modelling and validation of the weighted mean temperature for Turkey. Meteorol. Appl..

[26] Maghrabi A (2018). Variations and modeling of the atmospheric weighted mean temperature for ground-based GNSS applications: Central Arabian Peninsula. Adv. Space Res..

[27] Wang S, Xu T, Nie W, Wang J, Xu G (2020). Establishment of atmospheric weighted mean temperature model in the polar regions. Adv. Space Res..

[28] Wang M (2023). Region-specific and weather-dependent characteristics of the relation between GNSS-weighted mean temperature and surface temperature over China. Remote Sens..

[29] Zhang S (2023). A weighted mean temperature model using principal component analysis for Greenland. GPS Solut..

[30] Saxena S, Dwivedi R (2023). An ERA5 based local modelling of weighted mean temperature over hilly region in India for improved spatiotemporal analysis of extreme weather event using GNSS PWV. Adv. Space Res..

[31] Yang F (2024). Higher accuracy estimation of the weighted mean temperature (Tm) using GPT3 model with new grid coefficients over China. Atmos. Res..

[32] Landskron D, Böhm J (2018). VMF3/GPT3: Refined discrete and empirical troposphere mapping functions. J. Geodesy.

[33] Elhaty NM, Abdelfatah MA, Mousa AE, El-Fiky GS (2019). GNSS meteorology in Egypt: Modeling weighted mean temperature from radiosonde data. Alexandria Eng. J..

[34] Abdelfatah MA (2022). Artificial neural network for improving the estimation of weighted mean temperature in Egypt. J. Appl. Geodesy.

[35] Ma Y, Chen P, Liu T, Xu G, Lu Z (2021). Development and assessment of an ALLSSA-based atmospheric weighted mean temperature model with high time resolution for GNSS precipitable water retrieval. Earth Space Sci..

[36] ECMWF, the European Centre for Medium-Range Weather Forecasts. Accessed December, 2022. Website, https://cds.climate.copernicus.eu/cdsapp#!/home (2022).

[37] NOAA, National Oceanic and Atmospheric Administration. Accessed February, 2023. Website, https://ruc.noaa.gov/raobs/ (2023)

[38] Bevis M (1994). GPS meteorology: Mapping zenith wet delays onto precipitable water. J. Appl. Meteorol..

[39] Davis J, Herring T, Shapiro I, Rogers A, Elgered G (1985). Geodesy by radio interferometry: Effects of atmospheric modeling errors on estimates of baseline length. Radio Sci..

[40] Yao Y, Shan L, Zhao Q (2017). Establishing a method of short-term rainfall forecasting based on GNSS-derived PWV and its application. Sci. Rep..

[41] Long F, Hu W, Dong Y, Wang J (2021). Neural network-based models for estimating weighted mean temperature in China and adjacent areas. Atmosphere.

[42] Chen B (2018). Constructing a precipitable water vapor map from regional GNSS network observations without collocated meteorological data for weather forecasting. Atmos. Meas. Tech..

[43] Böhm J, Möller G, Schindelegger M, Pain G, Weber R (2015). Development of an improved empirical model for slant delays in the troposphere (GPT2w). GPS Solutions.

[44] He C (2017). A new voxel-based model for the determination of atmospheric weighted mean temperature in GPS atmospheric sounding. Atmos. Meas. Tech..

[45] Moore, D. S., and Kirkland, S. The basic practice of statistics (Vol. **2**): *WH Freeman New York* (2007).

[46] Sleem RE, Abdelfatah MA, Mousa AE, El-Fiky GS (2019). Performance analysis of the permanent and a regional GNSS networks in Egypt. Int. J. Sci. Eng. Res..

[47] Wang X, Zhang K, Wu S, Fan S, Cheng Y (2016). Water vapor-weighted mean temperature and its impact on the determination of precipitable water vapor and its linear trend. J. Geophys. Res. Atmos..

[48] Huang L, Liu L, Chen H, Jiang W (2019). An improved atmospheric weighted mean temperature model and its impact on GNSS precipitable water vapor estimates for China. GPS Solut..

